# The Problematic Online Dating Apps Use Scale (PODAUS): Development and evaluation of its psychometric properties

**DOI:** 10.1016/j.abrep.2024.100533

**Published:** 2024-02-04

**Authors:** Alessio Gori, Eleonora Topino, Mark D. Griffiths

**Affiliations:** a Department of Health Sciences, University of Florence, Via di San Salvi 12, Pad. 26, 50135 Florence, Italy; b Integrated Psychodynamic Psychotherapy Institute (IPPI), via Ricasoli 32, 50122 Florence, Italy; c Department of Human Sciences, LUMSA University of Rome, Via della Traspontina 21, 00193 Rome, Italy; d International Gaming Research Unit, Psychology Department, Nottingham Trent University, 50 Shakespeare Street, Nottingham NG1 4FQ, UK

**Keywords:** Problematic social media use, Social media addiction, Anxious attachment, Self-esteem, Fear of missing out, FoMO

## Abstract

The internet has facilitated significant transformations in interpersonal interactions, and offers new possibilities for finding romantic partners through the use of online dating apps. However, as with other technology-based tools, some individuals can develop problematic patterns of dating apps use, exhibiting symptoms resembling addiction. Consequently, the aim of the present study was the development of a new psychometric instrument to assess problematic dating apps use, the Problematic Online Dating Apps Use Scale (PODAUS), and the examination of its psychometric properties. The sample comprised 384 participants (254 females and 130 males; *M_age_*=25.90 years; *SD*=5.21) who used dating apps daily. They completed an online survey including the PODAUS, Bergen Social Media Addiction Scale, Cyber Pornography Addiction Test, Love Addiction Inventory-Short-Form, and Ten‐Item Personality Inventory. The PODAUS showed a one-factor structure with good indications of validity, reliability, and gender measurement invariance. Problematic online dating apps use was significantly associated with problematic social media use, problematic cyberpornography use, love addiction, and three personality traits (i.e., agreeableness, conscientiousness, and openness). The PODAUS is a new and succinct self‐report measure that assesses problematic dating apps use, and can be easily used in both research and clinical practice.

## Highlights

• The Problematic Online Dating Apps Use Scale (PODAUS) is a new self-report measure.
• The PODAUS could be used to assess problematic dating app use.
• PODAUS scores were associated with problematic social media use and problematic cyberpornography use.
• PODAUS scores were associated with love addiction.
• PODAUS scores were negatively related to Agreeableness, Conscientiousness and Openness.

## 1 Introduction

The internet has changed the way that individuals live their lives and has also provided the potential for connecting with other individuals, expanding social networks, and seeking romantic partners (Cotten et al., 2013). Within this context, dating apps have becoming increasingly popular and are used by millions of users worldwide (Statista, 2023a). Dating apps are software applications accessible on any internet-connected device, including smartphones, enabling users to create new personal connections, often with the goal of establishing personal, romantic, or sexual relationships (Castro & Barrada, 2020). At the time of writing, there were approximately 80 million online dating service users in Europe, with a forecast of constant growth for the next few years (Statista, 2023b). The reasons associated with the increasing use of this tool are varied. For instance, in Italy, where *Tinder* and *Badoo* are the most used online dating services (AppTweak, 2023) – and where the present study was conducted – motivations for online dating extend beyond seeking romantic partners. Individuals also use these platforms for chatting, socializing, and meeting new people. The primary motivators for engaging in online dating have been reported to be curiosity and the desire to cultivate new friendships (Statista, 2021). Online dating apps facilitate the search for individuals based on various preferred personal attributes, such as age, sex assigned at birth, and sexual orientation. Additionally, many of them leverage the global positioning system (GPS) to enhance connections among individuals in close proximity (see Anzani et al. [2018] for a review). As described by Chan (2017), dating apps possess five key advantageous characteristics: they can be effortlessly utilized in any location to identify individuals close to the user (*mobility*); they have the potential to facilitate quick encounters with individuals nearby (*proximity* and *immediacy*); users are often requested to register with an existing account, enhancing the likelihood of truthfulness (*authenticity*); and they place a strong emphasis on visual content, particularly images (*visual dominance*).

This technology is cost-effective, fast, user-friendly (Wiederhold, 2015), and has the potential to facilitate the formation of both short-term and long-term relationships (Danielsbacka, Tanskanen, & Billari, 2022), thereby mitigating feelings of loneliness (Sumter, Vandenbosch, & Ligtenberg, 2017). However, in addition to these positive effects, research has identified associations between the compulsive use of dating apps and offline interpersonal problems (Sharabi & Timmermans, 2021), as well as higher levels of psychological distress, and symptoms of anxiety and depression (Holtzhausen et al., 2020). A number of studies have identified that some individuals may struggle with controlling their online dating app usage (Orosz et al., 2018) and have suggested classifying problematic dating apps use as a behavioral addiction (Orosz et al., 2016, Orosz et al., 2016). This concurs with previous evidence regarding problematic use of other internet-based activities (see Pontes et al. [2015] for a review). However, research on problematic online dating apps use remains limited and warrants further investigation (Her & Timmermans, 2021).

### 1.1 Problematic online dating apps use: Definition and assessment tools

Using the components model of addiction (Griffiths, 2005), problematic online dating apps use may be conceptualized as the persistent and recurrent use of dating apps characterized by:

• *Salience:* The use of dating apps becoming a central role in an individual’s life and dominating their thoughts and behavior;
• *Mood modification:* Dating apps being used to modify the individual’s mood state;
• *Tolerance:* Over time, the individual needing to use dating apps more and more to have the same mood modifying effect;
• *Withdrawal:* Unpleasant feelings and psychological distress when not being able to use dating apps;
• *Conflict:* Dating apps use compromising social relationships and other important areas including occupation and/or education;
• *Relapse:* Returning to previous patterns of dating apps use after a period of abstinence.

Using this model, the Problematic Tinder Use Scale (Orosz et al., 2016, Orosz et al., 2016) was developed, which was the first study to explore problematic online dating app use. Subsequent research examined the factors associated with problematic *Tinder* use, showing the significant predictive role of personality traits, self-esteem, and relatedness need frustration (Orosz et al., 2018). Moreover, another study identified a profile of high-level problematic *Tinder* users, characterized by high levels of anxious attachment, sexual desire, urgency, and sensation-seeking, and a moderate level of self-esteem (Rochat et al., 2019). Recent research also indicated a significant and negative association between problematic *Tinder* use and safe sex behavior (Liberacka-Dwojak et al., 2023) and a significant and positive relationship between problematic *Tinde*r use, problematic social media use, and problematic online sexual behaviors (Harren et al., 2021).

However, the Problematic Tinder Use Scale focuses on one specific application (*Tinder*), whereas in recent years, many new dating apps have gained popularity (Coduto, Lee-Won, & Baek, 2020). Therefore, a focus on just *Tinder* could be restrictive in comprehensively addressing the phenomenon, in the present context. Other psychometric measures have focused on specific aspects of dating apps use, such as the Tinder Use Motivation Scale (Orosz et al., 2018) and the Tinder Motives Scale (Timmermans & De Caluwé, 2017a), which both focus on motivations to use *Tinder.* Other scales assess variables relating to online dating more generally (as opposed to dating apps specifically), focusing on intensity (i.e., Online Dating Intensity Scale [Bloom, & Dillman Taylor, 2020]), perceived quality (Cyberdating Q_A [Sánchez, Muñoz-Fernández, & Ortega-Ruíz, 2015]), and the type of use (Online Dating Inventory [Blackhart et al., 2014]).

It should also be noted that some previous studies have adapted scales dedicated to the problematic use of the internet in general or specific types of internet use (e.g., social media use) to assess the problematic use of dating apps. However, these lacked factorial analyses, information regarding discriminant validity, and/or any in-depth psychometric evaluation of these measures (e.g., Coduto et al., 2020, Hu, 2023, Hu and Rui, 2023). Moreover, to the best of the authors’ knowledge, there are currently no psychometrically-validated scales that assess general problematic online dating apps use using the components model of addiction as its theoretical underpinning (Griffiths, 2005).

### 1.2 The present study

Since dating apps are now a widely used tools (see Bonilla-Zorita et al., [2021] for a review), it is important to achieve a greater understanding of their problematic use to inform clinical practice and guide preventive activity. For this purpose, the presence of psychometrically solid and theoretically-founded assessment measures is necessary.

Based on the aforementioned considerations, the goal of the present study was to develop the Problematic Online Dating Apps Use Scale (PODAUS), a new self-report psychometric instrument to assess problematic dating apps use based on the components model of addiction (Griffiths, 2005). The specific aims were the development of the items and the evaluation of the psychometric properties of the scale. Moreover, since previous preliminary research showed the relationships between problematic *Tinder* use and (i) personality traits, (ii) romantic motivations, (iii) social motivations, and (iv) sexual motivations (Orosz et al., 2018, Timmermans and De Caluwé, 2017a), the associations between problematic dating apps use and problematic social media use, problematic cyberpornography use, love addiction, and personality traits were investigated to examine convergent and divergent validity.

## 2 Method

### 2.1 Participants

The study sample comprised 384 Italian participants (254 females and 130 males; *M_age_* = 25.90 years; *SD* = 5.21) who used online dating apps daily (see Table 1). Many of them reported that they had obtained a university degree (53 %), were currently students (47 %), and were not in a romantic relationship at the time of completing the survey (73 %). Regarding their daily use of dating apps, 72 % reported using them for up to 1 h, 21 % for 1 to 2 h, 5 % for more than 2 up to 5 h, 1 % for more than 5 up to 10 h, and less than 1 % for more than 10 h.

**Table 1:** Demographic characteristics of the sample (n = 384).

Characteristics		M ± SD	n	%
	Age	25.90 ± 5.21		

Gender				
	Males		130	33.9
	Females		254	66.1

Education				
	Elementary school		1	0.3
	Middle School diploma		10	2.6
	High School diploma		87	22.7
	University degree		203	52.9
	Master’s degree		68	17.7
	Post-lauream specialization		15	3.9

Professional Condition				
	Student		180	46.9
	Working student		110	28.6
	Retired		1	0.3
	Employee		62	16.1
	Manager		1	0.3
	Freelance		11	2.9
	Entrepreneur		2	0.5
	Trader		1	0.3
	Homemaker		3	0.8
	Unemployed		13	3.4

Romantic relationship				
	No		281	73.2
	Yes, less than a month		8	2.1
	Yes, from 1 months to less than 6 months		24	6.3
	Yes, from 6 months to less than a year		15	3.9
	Yes, from 1 year to less than 2 years		19	4.9
	Yes, from 2 years to less than 5 years		17	4.4
	Yes, from 5 years to less than 10 years		11	2.9
	Yes, for 10 years or more		9	2.3

Dating apps use (daily)				
	Up to 1 h		277	72.1
	From 1 up to 2 h		82	21.4
	More than 2 up to 5 h		20	5.2
	More than 5 up to 10 h		3	0.8
	More than 10 h		2	0.5

### 2.2 Procedure and ethics

The Problematic Online Dating Apps Use Scale (PODAUS) was developed by conceptualizing items based on the six core components in the addiction components model (Griffiths, 2005). Therefore, six items were developed (see Appendix A), each corresponding to one of the aforementioned addiction components (i.e., salience, tolerance, mood modification, relapse, withdrawal, and conflict). The authors engaged in discussions and restructured items to ensure clear and appropriate language, maintain theoretical consistency, and minimize ambiguity, until a satisfactory agreement was reached. Participants in the present study were recruited online using a snowball sampling procedure. More specifically, a link to the survey was disseminated through social networks (e.g., authors’ *Facebook* profiles) and instant messaging services (e.g., *WhatsApp* conversations) and asking potential participants to share it with others. Inclusion criteria were: (i) being at least 18 years old; (ii) having a good command of the Italian language; and (iii) using online dating apps daily. Prior to beginning the survey, participants were informed about the overall purpose of the study, and assurance was given regarding the protection of their privacy and anonymity. After providing electronic informed consent, they proceeded to complete the survey and a demographic questionnaire hosted on the *Google Forms* platform. All the procedures of the study were approved by the first author’s institutional Ethical Committee.

### 2.3 Measures

#### 2.3.1 Demographic questionnaire

Some general participant information was requested with questions concerning gender, age, education, current engagement in a romantic relationship (including its duration if applicable), and daily time spent using online dating apps.

#### 2.3.2 Problematic Online Dating Apps Use Scale (PODAUS)

The PODAUS is a six-item scale used to assess problematic dating apps use. The six items relate to each of the six different core components of addiction (Griffiths, 2005). Items are rated on a five-point Likert scale from 1 (*strongly disagree*) to 5 (*strongly agree*). The total score is calculated by adding the scores of each individual item with scores ranging from 6 to 30. The higher the score, the greater the risk of problematic online dating apps use. In the present sample, the scale showed good indications of internal consistency (for more details on the factor structure and the reliability of the PODAUS, see the Results section).

#### 2.3.3 Bergen Social Media Addiction Scale (BSMAS)

The BSMAS (Andreassen et al., 2016; Italian version: Monacis et al., 2017) is a six-item scale used to assess problematic social media use, based on the components model of addiction (Griffiths, 2005). Items are rated on a five-point Likert scale from 1 (*very rarely*) to 5 (*very often*). In the present study, the Italian BSMAS showed good internal consistency (*α* = 0.85; *ω* = 0.84).

#### 2.3.4 Cyber Pornography Addiction Test (CYPAT)

The CYPAT (originally developed in Italian by Cacioppo et al., [2018]) is an 11-item scale used to assess problematic cyberpornography use. Items are rated on a five-point Likert scale from 1 (*never*) to 5 (*always*). The higher the score, the greater the risk of problematic cyberpornography use. In the present study, the Italian CTPAT showed good internal consistency (*α* = 0.94; *ω* = 0.94).

#### 2.3.5 Love Addiction Inventory—Short-Form (LAI-SF)

The LAI–SF (originally developed in Italian by Costa et al., 2021) is a six-item scale used to assess love addiction, based on the components model of addiction (Griffiths, 2005). Items are rated on a five-point Likert scale from 1 (*never*) to 5 (*very often*). In the present study, the Italian LAI–SF showed good internal consistency (*α* = 0.90; *ω* = 0.90).

#### 2.3.6 Ten‐Item Personality Inventory (TIPI)

The TIPI (Gosling et al., 2003; Italian version: Di Fabio, Gori, & Giannini, 2016) is a 10-item scale used to assess the Big Five personality traits (Costa & McCrae, 1992). Items are rated on a seven‐point Likert scale, ranging from 1 (*disagree strongly*) to 7 (*agree strongly*). Five personality dimensions are assessed: extraversion, agreeableness, conscientiousness, neuroticism, and openness. The Italian version was used in the present research and showed acceptable internal consistency (extraversion, *α* = 0.77, *ω* = 0.76; agreeableness, *α* = 0.64, *ω* = 0.60; conscientiousness, *α* = 0.73, *ω* = 0.70; neuroticism, *α* = 0.61, *ω* = 0.60; openness *α* = 0.61; *ω* = 0.60).

### 2.4 Data analysis

The statistical analyses were performed using SPSS (IBM, Armonk, New York), AMOS (IBM, New York), and JASP (JASP Team, 2023) software. Descriptive statistics and item analysis were performed for each item of the PODAUS. An absolute skew value equal to or less than 2 and an absolute kurtosis equal to or less than 7 was considered indicative of normal distribution (Kim, 2013). The suitability of the data for factor analysis was evaluated by employing the Kaiser-Meyer-Olkin (KMO) statistic and Bartlett’s test of sphericity. A KMO > 0.7 and a statistically significant Bartlett’s test result (*p* < 0.001) were regarded as indicators of data appropriateness (Mulaik, 2009). To test the dimensionality of the PODAUS, the sample was randomly split into two subsamples. In the first one, an exploratory factor analysis (EFA) with a principal axis factoring extraction method (Promax rotation with Kaiser normalization) was performed, identifying the number of factors based on parallel analysis.

The factor structure was further tested implementing confirmatory factor analysis (CFA) in the second subsample, considering the following indices: the discrepancy divided by degree of freedom (*χ^2^*/DF), suggesting a reasonable fit for values <5 (Marsh & Hocevar, 1985); the Comparative Fit Index (CFI), Tucker Lewis index (TLI), and the Goodness of Fit (GFI) suggesting a reasonable fit for values >0.90 (Hu and Bentler, 1999, Kline, 2015); and the standardized root mean square residual (SRMR), suggesting a reasonable fit with values <0.08 (Hooper et al., 2008). Factor loadings exceeding 0.40 were considered indicative of a substantial item loading on a factor (Hair et al., 2018).

Measurement invariance across genders was assessed by examining three levels of invariance (configural, metric, and scalar) through a series of multigroup CFAs with progressively increasing constraints. The adopted criteria for support evidence of non-invariance included adequate goodness-of-fit indices for configural invariance, cutoffs of 0.01 for ΔCFI, paired with changes in SRMR of 0.030 for metric invariance or 0.015 for scalar or residual invariance (Chen, 2007).

Information about the reliability was investigated using item‐total correlation indices, alpha (Cronbach, 1951) and omega (McDonald, 2013) coefficients. To examine the associations between PODAUS and the variables used to explore some aspects of construct validity, Pearson’s correlation was carried out. Composite reliability (CR) and average variance extracted (AVE) values were computed to evaluate convergent validity, while the discriminant validity was assessed by calculating maximum shared variance (MSV) values and the heterotrait-monotrait (HTMT) ratio of correlations, using an AMOS plugin (Gaskin & James, 2019). The following conditions must be met to establish convergent validity: CR > 0.7, CR > AVE, and AVE > 0.5 (Hair et al., 2018). Concerning discriminant validity, MSV should be lower than AVE (Hair et al., 2018), and the HTMT ratio of correlations should not exceed the threshold of 0.85 (Henseler et al., 2015).

## 3 Results

Descriptive statistics of the sample are reported in Table 1. Absolute values of skewness and kurtosis were all less than 2 and 7, respectively (see Table 2), suggesting a normal distribution of the sample.

**Table 2:** Descriptive statistics and item‐total correlations of each PODAUS item.

Component	Item^a^	Mean^b^	*SD*	Skewness	Kurtosis	Item‐total correlation
Salience	1. I spend too much time using or thinking about dating apps.	1.854	1.108	1.126	0.173	0.723
Mood modification	2. I use dating apps as a way to change my mood (e.g., to escape, to feel better, etc.).	2.693	1.205	0.023	−1.128	0.422
Tolerance	3. Over time I have increased the amount of time I spend using or thinking about dating apps.	2.154	1.233	0.781	−0.490	0.667
Withdrawal	4. I become restless if I am unable to use dating apps.	1.484	0.873	1.918	3.186	0.754
Conflict	5. My use of dating apps interferes with important things in my life (e.g., education, occupation).	1.510	0.888	1.847	2.977	0.738
Relapse	6. I have tried to cut down my dating apps use but I have been unable to do it.	1.576	0.991	1.758	2.308	0.666

a English translation of the items from the original version (in Italian).

b Each item has a minimum score of 1 and a maximum score of 5.

**Fig. 1:**
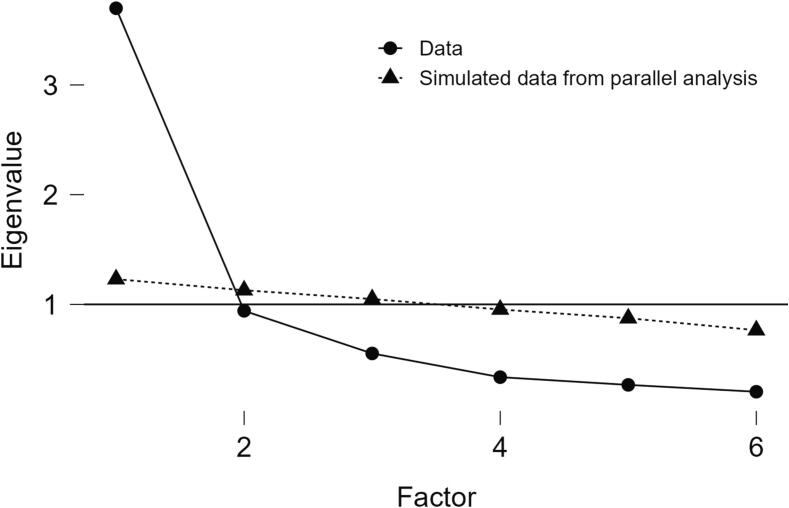
Scree plot.

A KMO value of 0.862 and the statistically significant value of Bartlett’s test supported the data suitability for factor analysis. The EFA showed a factor structure with one principal dimension with 70 % of the total variance explained (eigenvalue = 61.645), as shown in the scree plot (Fig. 1).

**Fig. 2:**
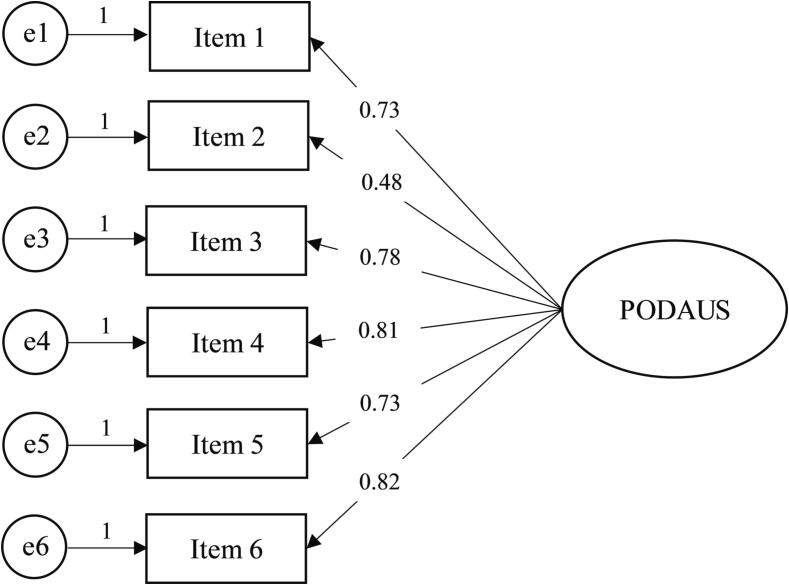
Confirmatory Factor Analysis for the one-factor structure of the PODAUS.

The CFA (see Fig. 2) demonstrated that the one-factor model provided a strong fit to the data, with all indices falling within the specified cutoff values: *χ^2^*/DF = 4.851, CFI = 0.943, TLI = 0.904, GFI = 0.975, and SRMR = 0.058. Moreover, measurement invariance across genders was confirmed (see Table 3).

**Table 3:** Results of measurement invariance testing across males and females.

	*χ^2^*/DF	CFI	TLI	GFI	SRMR	ΔCFI	ΔSRMR
Configural invariance	2.748	0.951	0.918	0.974	0.057		
Metric invariance	2.707	0.939	0.920	0.968	0.083	0.010	0.026
Scalar invariance	2.553	0.930	0.927	0.964	0.091	0.009	0.008

***Note:****χ^2^*/DF = the discrepancy divided by degree of freedom, CFI = Comparative Fit Index, TLI = Tucker Lewis index, GFI = Goodness of Fit, SRMR = standardized root mean square residual, ΔCFI = Difference in CFI values between the compared models, ΔSRMR = Difference in SRMR values between the compared models.

Concerning the reliability of the scale, the Cronbach alpha (*α* = 0.856) and McDonald’s omega (ω = 0.850) indices of PODAUS were good and the item total correlations (see Table 2) ranged from 0.422 (Item 2) to 0.754 (Item 4). Pearson’s correlation (see Table 4) showed that PODAUS scores were significantly and positively associated with BSMAS (*r* = 0.412, *p* < 0.01), CYPAT (*r* = 0.594, *p* < 0.01), and LAI-SF (*r* = 0.343, *p* < 0.01) scores. This suggests good convergent validity, which is further proven by the CR and AVE values, which met the required criteria (see Table 4). PODAUS scores were also significantly and negatively correlated with agreeableness (*r* = −0.181, *p* < 0.01), conscientiousness (*r* = −0.145, *p* < 0.01), and openness (*r* = −0.215, *p* < 0.01). Finally, the MSV value was lower than AVE one, and all the HTMT indices were below the threshold value of 0.85, supporting the absence of discriminant validity problems (see Table 4).

**Table 4:** Correlations (below the diagonal), HTMT, CR, AVE, and MSV (over the diagonal).

	1	2	3	4	5	6	7	8	9	CR	AVE	MSV
1. PODAUS	–	0.478	0.675	0.393	0.024	0.288	0.192	0.141	0.306	0.871	0.542	0.463
2. BSMAS	0.412**	**–**	0.420	0.401	0.094	0.146	0.259	0.235	0.082			
3. CYPAT	0.594**	0.376**	**–**	0.355	0.039	0.250	0.209	0.194	0.235			
4. LAI-SF	0.343**	0.348**	0.324**	**–**	0.033	0.272	0.210	0.281	0.215			
5.Extraversion (TIPI)	0.014	−0.081	−0.040	−0.027	–	0.232	0.102	0.115	0.415			
6. Agreeableness (TIPI)	−0.181**	−0.104*	−0.164**	−0.206**	0.130*	**–**	0.323	0.453	0.429			
7. Conscientiousness (TIPI)	−0.145**	−0.215**	−0.172**	−0.182**	0.085	0.194**	**–**	0.282	0.199			
8. Neuroticism (TIPI)	−0.100	0.168**	−0.146**	0.203**	−0.080	−0.292**	−0.196**	**–**	0.068			
9. Openness (TIPI)	−0.215**	−0.061	−0.176**	−0.161**	0.275**	0.229**	0.128*	−0.040	–			

HTMT = heterotrait-monotrait ratio of correlations; CR = composite reliability; AVE = average variance extracted; MSV = maximum shared variance.

** Correlation is significant at the *p* < 0.01 level (2-tailed).

* Correlation is significant at the *p* < 0.05 level (2-tailed).

## 4 Discussion

The expansion of online activities and the emergence of real-time location-based dating apps have opened up novel avenues for meeting individuals and establishing relationships with potential romantic partners (Castro & Barrada, 2020). Nevertheless, akin to other online entertainment and socialization activities, such as internet gaming (American Psychiatric Association, 2013, American Psychiatric Association, 2022) and social media use (Andreassen et al., 2016, Gori et al., 2023a), the utilization of online dating apps also appears to lead to potentially addictive behaviors. Therefore, the development of psychometric scales that can evaluate problematic use of online dating apps may be useful and beneficial for both clinical practice and research, to promote a better assessment and understanding of the phenomenon. With this rationale in mind, the present study developed the Problematic Online Dating Apps Use Scale (PODAUS), a new self-report instrument to assess problematic online dating apps use, and investigated its psychometric properties.

The process of generating items was guided by the theoretical framework of the addiction components model (Griffiths, 2005). This approach is consistent with the development of numerous tools used to assess various behavioral addictions, such as exercise addiction (Terry et al., 2004, Gori et al., 2023b) and sex addiction (Andreassen et al., 2018, Soraci et al., 2023). It also aligns with other psychometric scales assessing different forms of problematic online behaviors, such as problematic social media use (Bergen Social Media Addiction Scale; Andreassen et al., 2016), problematic series watching (Problematic Series Watching Scale; Orosz et al., 2016, Orosz et al., 2016), online shopping addiction (Bergen Shopping Addiction Scale; Andreassen et al., 2015), problematic *QQ* use (Problematic QQ Use Scale; Liu et al., 2021), and mukbang addiction (Mukbang Addiction Scale; Kircaburun et al., 2021). This process resulted in a self‐report scale comprising six items, one for each addiction component (see Appendix A and Table 2 for the original version and English translation of the items relating to salience, tolerance, mood modification, relapse, withdrawal, and conflict).

The PODAUS had excellent psychometric properties, with good indications of validity and reliability. The EFA indicated a clear factor structure characterized by a single dimension that accounted for a significant proportion of the variance (i.e., 70 %). This finding, which was further substantiated by the CFA, aligns with other brief psychometric measurement tools based on the addiction components model (e.g., Andreassen et al., 2018, Orosz et al., 2016, Orosz et al., 2016, Terry et al., 2004). Evidence for cross-gender invariance was also found, supporting the psychometric equivalence of the PODAUS scores for males and females. Moreover, although each PODAUS item related to a different addiction component, the scale showed good internal consistency. This provides support for the robust psychometric properties of this instrument and its reliability in evaluating problematic online dating apps use.

The results also showed a significant and positive association between PODAUS scores and the variables used to assess convergent validity, while simultaneously displaying scores that were clearly distinguishable from them, therefore suggesting good discriminant validity. More specifically, problematic online dating apps use was significantly and positively associated with problematic cyberpornography use. This is in line with previous evidence highlighting the significance of sexual motivation as a predictor of problematic online *Tinder* use (Orosz et al., 2018), which, in turn, has been found to be associated with problematic online sexual behaviors (Harren, Walburg, & Chabrol, 2021).

Additionally, evidence has consistently shown a significant relationship between the dysregulated use of online dating apps and a higher risk of engaging in risky sexual behaviors (see Bonilla-Zorita et al. [2021] for a review). Not surprisingly, PODAUS scores were also significantly and positively associated with love addiction. Furthermore, previous studies have shown that romantic motivation is associated with problematic *Tinder* use (Orosz et al., 2018). Indeed, online dating apps serve as a platform to initiate relationships that can culminate in face-to-face encounters (Alam, Yeow, & Loo, 2011) and fulfil the desire to establish a romantic relationship (Timmermans & De Caluwé, 2017b).

Moreover, the results also highlighted a significant and positive association between problematic online dating apps use and problematic social media use, concurring with previous research showing a significant and positive relationship between problematic *Tinder* use and problematic social media use (Harren et al., 2021). Online dating apps are not only used to seek sexual pleasure or find a romantic partner but also to facilitate the formation of new friendships (Sumter et al., 2017). On the other hand, social media is sometimes used to search for romantic partners (Fox et al., 2014). Consequently, the use of these two types of platforms may share some common motivations (Harren et al., 2021).

With regards to personality traits, agreeableness was significantly and negatively related to PODAUS scores. To interpret such a result, it should be noted that the main motivations for using dating apps is the search for face-to-face relationships to satisfy sexual, friendship, or romantic needs (Timmermans & De Caluwé, 2017a). Therefore, individuals with higher levels of agreeableness may be able to achieve these objectives with greater ease (Tov et al., 2016), consequently perceiving lower need to rely on these platforms and, therefore, limiting the risk of developing problematic use of online dating apps. Moreover, PODAUS scores were also significantly and negatively related to conscientiousness. This is consistent with previous research showing that individuals with dysregulated *Tinder* use showed higher levels of perceived urgency, sensation seeking, and a lack of conscientiousness (Rochat et al., 2019). Finally, a significant and negative relationship was also found between PODAUS scores and openness. This finding aligns with previous research on the motives for using online dating apps, which suggested that individuals with this personality trait are less inclined to use *Tinder* to relieve boredom and, presumably, are more likely to find other activities that reduce the possibility of getting bored (Orosz et al., 2018).

The present study has some limitations. First, the snowball sampling method may have limited generalizability due to its non-random nature. To address this limitation, future research could use mixed sampling methods, combining snowball sampling with random or stratified sampling techniques. Second, the sample predominantly comprised females, and this might also limit the generalizability of findings compared to a more gender-balanced population. To have a more comprehensive understanding of the phenomenon across various demographics, future research should recruit a more diverse and representative sample by employing targeted recruitment strategies or oversampling underrepresented groups. Moreover, the study relied solely on self-report measures, which may introduce potential biases and social desirability effects. A combination of self-report measures and objective assessments, such as behavioral observations (e.g., tracking user engagement and interaction patterns within dating apps) or physiological data (e.g., heart rate variability during app usage), should be considered in future research. In addition, problematic dating use is a relatively new and complex phenomenon, which deserves further explorations regarding its key features and associated factors (e.g., ‘swiping’, Thomas et al., 2023). Similarly, information regarding contextual use-trends, stigma, and/or dating app preferences was not collected. Such factors should be investigated in future research to examine their association with problematic online dating app use.

## 5 Conclusions

Research on problematic online dating apps use is in its nascent phase, despite the widespread popularity and use across the globe, irrespective of gender, age, sexual orientation, and other sociodemographic factors (Castro & Barrada, 2020). To help further research on this field, the present study developed the Problematic Online Dating Apps Use Scale (PODAUS), a new psychometric instrument to evaluate problematic dating apps use, based on the components model of addiction (Griffiths, 2005). The PODAUS demonstrated psychometrically robust properties and was theoretically underpinned using a model that has been used in the development of numerous scales to assess various behavioral addiction. This self-report scale can easily be used for screening in both research and clinical practice. Indeed, the availability of a measure for evaluating problematic online dating apps use could stimulate the examination of psychosocial factors associated with this behavior, broadening knowledge of this phenomenon and furnishing valuable insights to develop tailored interventions.

## Role of Funding Sources

This research did not receive any specific grant from funding agencies in the public, commercial, or not-for-profit sectors.

## Ethical Approval

All procedures performed in studies involving human participants were in accordance with the ethical standards of the Ethical Committee of the Integrated Psychodynamic Psychotherapy Institute (IPPI) (IPPI; ethical approval number 002/2023) and with Declaration of Helsinki of 1975, revised in 2013. Informed consent was obtained from all patients for being included in the study.

## CRediT authorship contribution statement

**Alessio Gori:** Conceptualization, Methodology, Formal analysis, Data curation, Writing – original draft, Writing – review & editing, Supervision. **Eleonora Topino:** Methodology, Formal analysis, Data curation, Writing – original draft, Writing – review & editing. **Mark D. Griffiths:** Conceptualization, Writing – original draft, Writing – review & editing, Supervision.

## Declaration of competing interest

The authors declare the following financial interests/personal relationships which may be considered as potential competing interests: Given his role as an Editorial Board member, M.D. Griffiths had no involvement in the peer-review of this article and had no access to information regarding its peer-review. All other authors have declared no conflicts of interest.

## Figures and Tables

**Table:** 

*Fortemente in disaccordo*	*Abbastanza in disaccordo*	*Né d’accordo né in disaccordo*	*Abbastanza d’accordo*	*Fortemente d’accordo*
**1**	**2**	**3**	**4**	**5**

**Table:** 

1. Trascorro troppo tempo usando o pensando alle dating app.	1	2	3	4	5
2. Uso le dating app per cambiare il mio umore (ad esempio, per evadere, per sentirmi meglio, ecc.).	1	2	3	4	5
3. Nel corso del tempo ho aumentato la quantità di tempo che trascorro utilizzando o pensando alle dating app.	1	2	3	4	5
4. Divento irrequieto/a se per qualche motivo non posso usare le dating app.	1	2	3	4	5
5. Il mio uso delle dating app interferisce con alcune aree importanti della mia vita (ad es. istruzione, occupazione).	1	2	3	4	5
6. Ho provato a ridurre l’utilizzo delle dating app ma non ci sono riuscito/a.	1	2	3	4	5

## Data Availability

The data that has been used is confidential.

## Appendix A

**Problematic Online Dating Apps Use Scale – Italian version**

Per favore, legga attentamente le seguenti affermazioni ed indichi il suo grado di accordo con esse, considerando la seguente scala:
